# Measles Virus Ribonucleoprotein Complexes Rapidly Spread across Well-Differentiated Primary Human Airway Epithelial Cells along F-Actin Rings

**DOI:** 10.1128/mBio.02434-19

**Published:** 2019-11-26

**Authors:** Brajesh K. Singh, Christian K. Pfaller, Roberto Cattaneo, Patrick L. Sinn

**Affiliations:** a Stead Family Department of Pediatrics, Carver College of Medicine, The University of Iowa, Iowa City, Iowa, USA; b Paul-Ehrlich-Institute, Division of Veterinary Medicine, Langen, Germany; c Department of Molecular Medicine, Mayo Clinic, Rochester, Minnesota, USA; Icahn School of Medicine at Mount Sinai

**Keywords:** actin, airways, lungs, measles, paramyxovirus

## Abstract

Measles virus (MeV) is a highly contagious human pathogen that continues to be a worldwide health burden. One of the challenges for the study of MeV spread is the identification of model systems that accurately reflect how MeV behaves in humans. For our studies, we use unpassaged, well-differentiated primary cultures of airway epithelial cells from human donor lungs to examine MeV infection and spread. Here, we show that the main components of the MeV ribonucleoprotein complex (RNP), the nucleocapsid and phosphoprotein, colocalize with the apical and circumapical F-actin networks. To better understand how MeV infections spread across the airway epithelium, we generated a recombinant virus incorporating chimeric fluorescent proteins in its RNP complex. By live cell imaging, we observed rapid movement of RNPs along the circumapical F-actin rings of newly infected cells. This strikingly rapid mechanism of horizontal trafficking across epithelia is consistent with the opening of pores between columnar cells by the viral membrane fusion apparatus. Our work provides mechanistic insights into how MeV rapidly spreads through airway epithelial cells, contributing to its extremely contagious nature.

## Abstract (precis)

The ability of viral particles to directly spread cell to cell within the airways without particle release is considered to be highly advantageous to many respiratory viruses. Our previous studies in well-differentiated, primary human airway epithelial cells suggest that measles virus (MeV) spreads cell to cell by eliciting the formation of intercellular membrane pores. Based on a newly generated ribonucleoprotein complex (RNP) “tracker” virus, we document by live-cell microscopy that MeV RNPs move along F-actin rings before entering a new cell. Thus, rather than diffusing through the cytoplasm of a newly infected columnar cell, RNPs take advantage of the cytoskeletal infrastructure to rapidly spread laterally across the human airway epithelium. This results in rapid horizontal spread through the epithelium that does not require particle release.

## INTRODUCTION

Measles virus (MeV), a highly transmissible human pathogen (1, 2), is a nonsegmented negative-strand RNA virus of the genus *Morbillivirus* in the family *Paramyxoviridae* (3). Its genome is organized into six transcription units and is enclosed by the nucleocapsid (N) protein, forming a ribonucleoprotein (RNP). The RNA-dependent RNA polymerase, constituted by a single L protein and minimally a homotetramer of the P protein, further contribute to the RNP complex (3). Viral particle assembly depends on the matrix (M) protein (4, 5), which also controls the activity of the membrane fusion apparatus that consists of the fusion (F) and hemagglutinin (H) proteins (6). In purified virus particles, the M protein can also form a helical layer around the RNP (7).

To enter the host and spread between immune cells, MeV takes advantage of the signaling lymphocytic activation molecule (SLAM; also known as CD150), which is expressed on alveolar macrophages and dendritic cells as well as in activated lymphocytes (8–10). Then, entry in the airways relies on the adherens junction protein Nectin-4, which is expressed at high levels in the columnar epithelial cells of the upper airways (11, 12).

Studies show that MeV remains largely cell associated. In the blood of humans or experimentally infected monkeys, no cell-free virus is detected; infections are detected by overlaying leukocytes onto SLAM-expressing cells (13, 14). In respiratory tract secretions, the amounts of cell-free MeV are low compared to other respiratory viruses (15). The ratio of intracellular to secreted MeV infectivity is about 10:1 in cultured cells (16). Particle-like MeV structures are reported to remain associated with the plasma membrane (17). These observations suggest that MeV may remain mainly cell associated during host infection.

Direct cell-to-cell spread offers a virus multiple advantages over release into the extracellular environment, such as efficiency, speed, and immune evasion (18, 19). Cell-associated spread is more efficient than particle formation because genomic cargo is delivered directly to a neighboring cell and fewer genomes are needed to start a productive infection. In addition, spread can be faster cell to cell because of the appropriation and modification of protein trafficking infrastructure (20). Lastly, direct cell-to-cell spread can sidestep intrinsic immunity and other barriers interfering with entry or postentry steps in target cells, and limited exposure time to the extracellular space allows for evasion of neutralizing antibodies (21).

Towards understanding the mechanisms of MeV spread, we use well-differentiated primary airway epithelial cells from human donors (HAE cells). These pseudostratified columnar epithelial cell sheets are comprised of multiple cell types, including ciliated, nonciliated, goblet, and basal cells (22). Using HAE cells, we demonstrated that: (i) MeV has a clear preference for basolateral entry (23); (ii) MeV infection results in the formation of infectious centers, not syncytia (24); and (iii) the transepithelial resistance remains intact for weeks after infection (24). Using MeV expressing green fluorescent protein (GFP), we observed that cytosolic GFP rapidly flows from infected into adjacent cells, suggesting the formation of pores along the lateral membrane of columnar epithelial cells, providing a route for direct cell-to-cell spread (24).

In the present study, we sought to determine how MeV proteins and RNPs traffic within and between cells. Within infected cells, N and P proteins localize to perinuclear replication centers and apical actin networks. We also focused on transmission from infected cells to neighboring naive cells. By live cell imaging, we observed rapid RNP movement along the apical filamentous actin (F-actin) rings, allowing viral spread to a new cell.

## RESULTS

### F-actin disassembles within infectious centers.

**FIG 1:**
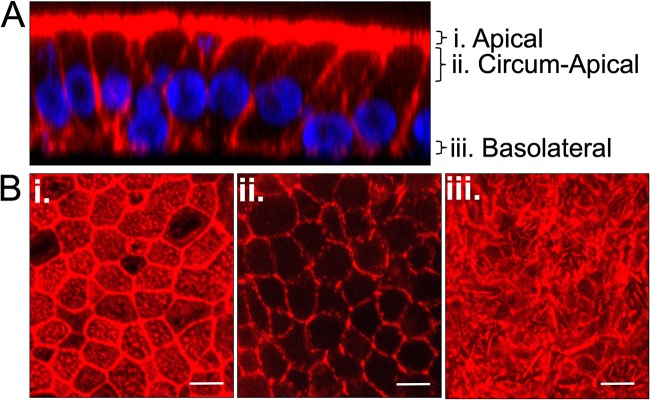
F-actin network in HAE cells. HAE cells were fixed and permeabilized, and F-actin filaments were visualized by staining with phalloidin (red) and nuclei by staining with DAPI (blue). Z-stacks were acquired by confocal microscopy. (A) The vertical section (*xz*) view from a z-stack series is shown. (B) The planes of the corresponding *en face* (*xy*) images are indicated. The F-actin networks in apical (i), circumapical (ii), and basolateral (iii) HAE cells are shown. As indicated, the *en face* views are comprised of maximum-intensity projection images of three to five z-stacks. Images are representative from *n* = 6 samples (two technical replicates from three human donors [biological replicates]). Scale bar, 20 μm.

In HAE cells, F-actin forms three distinct structural networks: apical, circumapical, and basolateral (Fig. 1). The apical network is a mesh of F-actin below the apical membrane that forms a complex with the nonmuscle myosin II cytoskeletal superstructure (25, 26) (Fig. 1Bi). The circumapical network is a thick circumferential belt composed of actin filaments encircling the uppermost portion of the lateral margins of epithelial cells (Fig. 1Bii). Indeed, most columnar epithelial cells have a dense belt of F-actin bundles at the level of the adherens junctions, where the MeV receptor Nectin-4 resides (24). This network is functionally tied to cell-cell adhesion and the formation of tight junctions (27–30). The basolateral network overlies the basolateral membrane and provides a support structure involved in cell-cell and cell-extracellular matrix adhesion (Fig. 1Biii).

**FIG 2:**
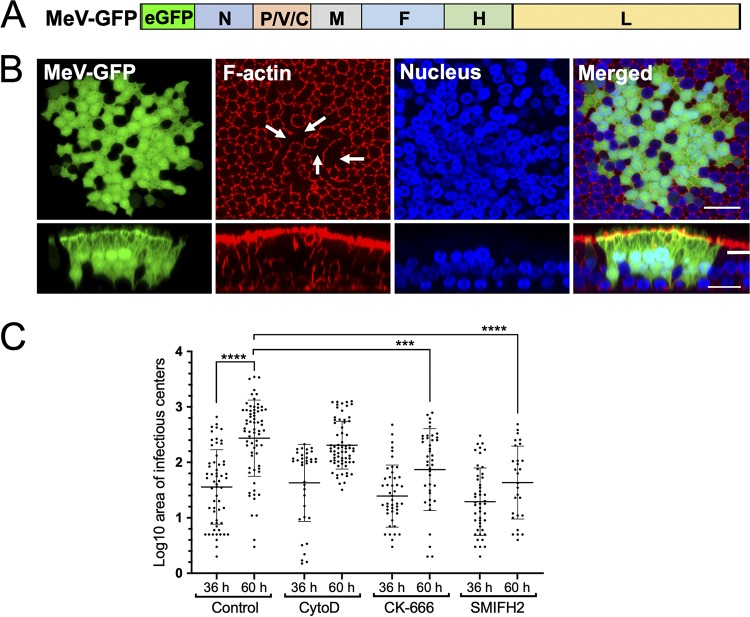
Structure of F-actin within infectious centers. (A) Schematic of the MeV-GFP genome. The eGFP coding region was inserted between the leader sequence and the N gene as a separate transcription unit. (B) HAE cells were infected with MeV-GFP (MOI = 1) and imaged using confocal microscopy 72 h later. Fixed and permeabilized HAE cells were stained for F-actin with rhodamine-conjugated phalloidin (red), and the nuclei were visualized with DAPI (blue). Both *en face* (upper panels) and vertical (lower panels) sections are shown. Arrows indicate the central regions of the infectious center where F-actin has disassembled. Scale bar, 20 μm. Images are representative from *n* = 6 samples (two technical replicates from three human donors [biological replicates]). (C) HAE cells were infected with MeV-GFP at an MOI of 1 and, 36 h later, the cultures were treated with the F-actin-disrupting agents cytochalasin D (CytoD), CK-666, or SMIFH2 or a control (DMSO) for 24 h. The areas of the infectious centers were quantified using ImageJ software at the time of drug delivery (36 h) and 24 h after drug delivery (60 h). Each dot represents the log-transformed value of the area of individual infectious centers. The data for each condition are pooled from six human donors. AU, arbitrary unit. Adjusted *P* value using one-way ANOVA corrected for multiple comparisons were determined (***, *P* < 0.001; ****, *P* < 0.0001).

Virus infection can lead to dynamic changes in the F-actin cytoskeleton (31–34), and previous studies show that the actin cytoskeleton impacts MeV infection in nonpolarized cells (17, 35, 36). Here, we used a recombinant MeV expressing GFP from an additional transcription unit (Fig. 2A) to characterize the progression of MeV infection in HAE. Three days after inoculation, the F-actin along the lateral margins and the circumapical network disassembled in the center of infectious centers, but remained readily detectable in cells at the periphery (Fig. 2B, *en face* views, and Video S1 in the supplemental material). Because infectious centers form by radiating out from an initial infected cell (23, 24, 37), we postulate that peripheral cells are more recently infected than central cells. Unlike the F-actin along the lateral margins, the apical and basolateral F-actin networks remained readily detectable by phalloidin staining in all cells of infectious centers, including centrally located cells (Fig. 2B, Video S1). This observation was consistent for every infectious center from every donor tested.

10.1128/mBio.02434-19.7 VIDEO S1 Lateral F-actin networks are disassembled within an infectious center. All confocal z-stacks of the infectious center (Fig. 2B) are shown from the apical to the basolateral surface. Z-stacks of 1 μm were acquired on a Leica SPE confocal microscope. HAE cells were infected with MeV expressing GFP (green). At 72 hpi, the cells were fixed, permeabilized, and incubated with rhodamine-conjugated phalloidin to stain for F-actin (red), and the nuclei were visualized with DAPI (blue). Download Movie S1, AVI file, 3.3 MB. Copyright © 2019 Singh et al. 2019 Singh et al. This content is distributed under the terms of the Creative Commons Attribution 4.0 International license.

To assess whether and how actin dynamics affects MeV spread, we treated HAE with three inhibitors of actin polymerization. Cytochalasin D (CytoD) binds the F-actin polymer and prevents further polymerization of monomers. CK-666 binds to the Arp2/3 protein complex that nucleates branched actin filaments (38). The small molecule inhibitor of formin homology 2 (SMIFH2) disrupts formin-dependent, but not Arp2/3 complex-dependent, actin cytoskeletal structures (39). The impact of the inhibitors on MeV spread in HAE was quantified by measuring the area of the infectious centers.

As shown in Fig. 2C, at the time of inhibitor application (36 h postinfection [hpi]), the sizes of the infectious centers were equivalent. Over the next 24 h (60 hpi), infectious center growth was significantly reduced in the CK-666 and SMIFH2 drug treatments compared to the dimethyl sulfoxide (DMSO) control. Of note, the SMIFH2 drug treatment had the greatest impact on infectious center growth, although the infectious center sizes after a 24-h treatment of CK-666 and SMIFH2 were not significantly different from each other. These findings potentially suggest a role for dynamic actin polymerization in the spread of MeV in HAE cells and may point to a specific role for formins.

### N, P, and M proteins localize near the apical and circumapical F-actin networks.

**FIG 3:**
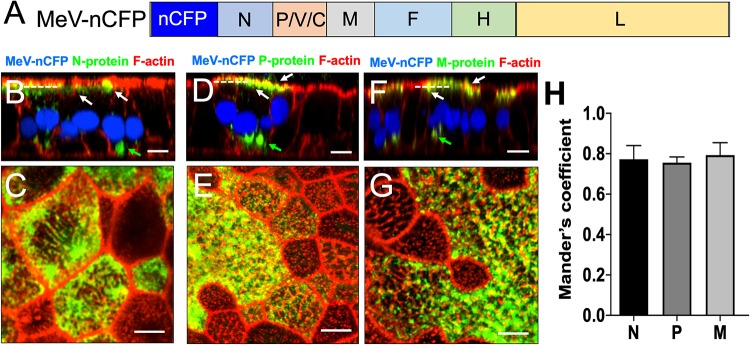
Localization of MeV N, P, and M proteins within infectious centers. (A) Schematic of the MeV-nCFP genome. The coding region of nuclear-targeted cyan fluorescent protein (nCFP) was inserted between the leader sequence and the N gene as a separate transcription unit. (B to G) Images of N, P, and M protein localization. HAE cells were infected with MeV-nCFP (blue) and, at 72 hpi, the cells were fixed and immunostained for N protein (B and C), P protein (D and E), or M protein (F and G) (green). F-actin was stained with phalloidin (red). The cells were examined by confocal microscopy. White arrows indicate apical localization, and green arrows indicate perinuclear localization. Vertical sections of immunostained cultures are shown in panels B, D, and F. Scale bar, 20 μm. Cells were then examined by stimulated emission depletion (STED) superresolution microscopy (C, E, and G). *En face* views of immunostained cultures are shown. Scale bar, 5 μm. Dotted lines in panels B, D, and F indicate the approximate plane of view for panels C, E, and G, respectively. Images are representative from *n* = 9 samples (three technical replicates from three human donors [biological replicates]). (H) Quantification of colocalization between viral proteins (N, P, and M) and F-actin at the apical surface in infectious centers was measured by applying Mander’s colocalization coefficient using Coloc2 plugin in Fiji.

Previous studies of MeV-infected immortalized cell lines suggest that F-actin is involved in particle assembly (40, 41). To characterize this process in HAE cells, we used the recombinant virus MeV-nCFP (Fig. 3A). This virus expresses a variant of the cyan fluorescent protein that includes a nuclear localization signal (nCFP), allowing not only immediate identification of infected cells but also interference-free study of cytoplasmic assembly processes (42). Cells were fixed and permeabilized 3-days postinfection before labeling with antibodies against N (Fig. 3B and C; see also Fig. S1A in the supplemental material), P (Fig. 3D and E; see also Fig. S1B), or M (Fig. 3F and G; see also Fig. S1C). F-actin was stained in red by phalloidin.

10.1128/mBio.02434-19.1 FIG S1 Localization of MeV proteins in infectious centers. HAE cells were infected with MeV-nCFP (blue), counterstained for F-actin using phalloidin (red) or either of MeV proteins N protein (A), P protein (B), and M-protein (green) (C) and imaged by confocal microscopy. The left panel shows vertical sections that correspond to the same vertical sections shown in Fig. 3. The right panels are different planes on its *z* axis, as indicated by the vertical bars. The right panel shows maximum intensity projection images of three to five z-stacks at the indicated apical and circumapical regions. The viral proteins primarily colocalize with F-actin near the apical surface of HAE. The green arrows indicate the reproducible lack of the circum-apical actin network at the center of infectious centers. The white arrows indicate viral protein association with F-actin. Images are representative from *n* = 9 (three technical replicates from three human donors [biological replicates]). Scale bars, 20 μm. Download FIG S1, PDF file, 2.5 MB. Copyright © 2019 Singh et al. 2019 Singh et al. This content is distributed under the terms of the Creative Commons Attribution 4.0 International license.

For both N and P proteins, we monitored two distinct sites of accumulation in HAE cells. N and P were readily detectable in large perinuclear bodies (Fig. 3B and D; see also Fig. S1A and B). These bodies may be the sites of viral transcription and replication (43, 44). In addition, proximal to the apical plasma membrane, localization was observed (Fig. 3B and D; see also Fig. S1A and B). These sites colocalize with the apical F-actin network. M protein was also occasionally found in perinuclear bodies (Fig. 3F; see also Fig. S1C) but accumulated mainly at the apical membrane.

We then used stimulated emission depletion (STED) microscopy to examine the extent of colocalization of the viral proteins N (Fig. 3C), P (Fig. 3E), and M (Fig. 3G) with the apical F-actin network at higher resolution. Again, we used MeV-nCFP, whose fluorescent reporter protein is segregated to the nucleus. We quantified the colocalization of MeV proteins to apical F-actin by Mander’s colocalization coefficient (MCC) analysis (Fig. 3H). This analysis of N, P, and M proteins (green) and F-actin (red) signals in dually labeled images resulted in MCC values of 0.773 ± 0.07, 0.755 ± 0.03, and 0.793 ± 0.06, respectively. A value of 1 is perfectly colocalized, and a value of −1 is perfectly mislocalized. These data confirm the close proximity of all three viral proteins to apical F-actin.

### Generation and validation of recombinant MeV-RNP^tracker^.

**FIG 4:**
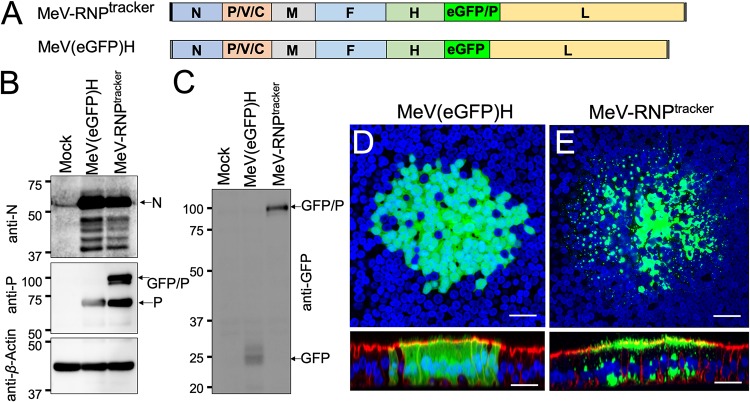
Generation and characterization of MeV-RNP^tracker^. (A) Schematics of the MeV-RNP^tracker^ genome (top) and of the MeV(GFP)H genome (bottom) control virus. In MeV-RNP^tracker^, GFP was fused in frame with a second copy of the P protein (GFP/P), and the transcription unit was inserted between the H and L genes. In MeV(GFP)H, a transcription unit expressing GFP was inserted in the same position. (B and C) Immunoblot characterization of the P proteins expressed by the two viruses. In panel B, the expression of the N (top), P and GFP/P (center), or control actin (bottom) proteins was analyzed. In panel C, the expression of GFP/P and GFP proteins were analyzed. At 3 days postinfection, the GFP expression of MeV(GFP)H (D) and MeV-RNP^tracker^ (E) was examined by confocal microscopy. Nuclei were stained with DAPI (blue), and F-actin was visualized with phalloidin (red, only shown in the bottom panel vertical sections). Images are representative from *n* = 6 samples (two technical replicates from three human donors [biological replicates]). Scale bars, 20 μm.

We previously monitored the intercellular transport of cytoplasmic GFP in MeV-infected HAE cells (24). To follow the intercellular spread of MeV RNP in real time, we generated here a recombinant MeV that incorporates GFP in its RNP, termed MeV-RNP^tracker^. In addition to the standard P protein, this virus expresses a GFP/P fusion protein from an additional transcription unit inserted between the H and L genes (Fig. 4A). The P and GFP/P proteins are expressed at similar levels (Fig. 4B and C). As a control, we also generated MeV(GFP)H, which expresses soluble GFP from the same transcriptional unit between H and L; this virus differs from MeV-GFP (Fig. 2A) only by the location of the GFP transcriptional unit within the MeV genome. Both recombinant MeV(GFP)H and RNP^tracker^ are viable, and their replication kinetics are similar in Vero-hSLAM cells (Fig. S2A), but the replication kinetics of RNP^tracker^ were slightly delayed in an epithelial cell line H358 (Fig. S2B). We further compared their replication in HAE cells. Infection of HAE cells with RNP^tracker^ results in similar numbers and sizes of infectious centers compared to MeV(GFP)H (Fig. S2C and D).

10.1128/mBio.02434-19.2 FIG S2 Characterization of MeV-RNP^tracker^. Growth kinetics of recombinant MeVs in Vero-hSLAM cells (A) and in epithelial cell line H358 cells (B) are shown. Cells were infected with MeV at an MOI of 0.01. At various time points, the cells were harvested, and the TCID_50_/ml were determined. The data represent the means ± the standard deviations of results from triplicate experiments. The solid and dashed lines indicate data for MeV(GFP)H and RNP^tracker^ virus titers, respectively. HAE were infected with MeV(GFP)H or RNP^tracker^ at an MOI of 1 and, 72 h later, images were acquired using an inverted florescence microscope. The numbers (C) and areas (D) of infectious centers were determined using ImageJ software. Images are representative from *n* = 6 (two technical replicates from three human donors [biological replicates]). Download FIG S2, PDF file, 0.10 MB. Copyright © 2019 Singh et al. 2019 Singh et al. This content is distributed under the terms of the Creative Commons Attribution 4.0 International license.

We then monitored the spread of these two viruses in HAE cells. The infectious centers of MeV(GFP)H (Fig. 4D) were characterized by diffuse cytoplasmic fluorescence, indistinguishable from what was observed with MeV-GFP (Fig. 2B). In contrast, in the MeV-RNP^tracker^ infectious centers, GFP signals were punctate, concentrated along the intracellular surface of the apical membrane, and in perinuclear regions (Fig. 4E). These observations, which recapitulate the immunohistochemistry results using anti-P and anti-N antibodies in MeV-GFP-infected HAE cells (Fig. 3), are consistent with incorporation of the GFP/P protein in RNPs.

**FIG 5:**
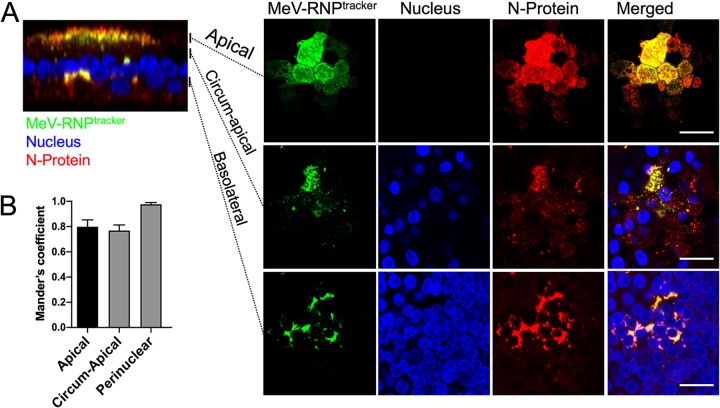
Intracellular distribution of RNPs in RNP^tracker^ infected HAE. (A) Cells were infected with MeV-RNP^tracker^ and imaged at 72 hpi by confocal microscopy. The cells were fixed, permeabilized, and then immunostained for N protein (red). Nuclei were visualized with DAPI (blue). The left panel is a vertical section view, and the vertical bars indicate the plane of view for the series of *en face* images on the right. The right panels show maximum intensity projection images of three to five z-stacks at the indicated apical, circumapical, and basolateral regions. Scale bars, 20 μm. Images are representative from *n* = 6 samples (two technical replicates from three human donors [biological replicates]). (B) Colocalization between RNP^tracker^ and N protein within infectious centers was measured by using Mander’s colocalization coefficient.

To confirm that GFP-tagged P protein produced from RNP^tracker^ is incorporated into the RNP complex, we immunostained for P and N in RNP^tracker^-infected HAE cells (Fig. 5A and Fig. S3A). Indeed, the GFP-tagged P protein colocalized with the N and P proteins at apical, circumapical, and perinuclear regions of infectious centers (Fig. 5, Fig. S3, and Video S2 and S3). MCC analysis of overlap for RNP^tracker^ (green) with N (Fig. 5B) and P (Fig. S3B) proteins (red) showed strong colocalization at apical, circumapical, and perinuclear regions within infectious centers. These data validate the use of MeV-RNP^tracker^ to characterize the intercellular transport of MeV RNPs.

10.1128/mBio.02434-19.3 FIG S3 Localization of RNP^tracker^ and P protein in infectious centers. (A) HAE cells were infected with MeV-RNP^tracker^ (green). At 72 hpi, the cells were fixed, permeabilized, and colabeled using anti-P-protein antibodies (red), and nuclei were visualized with DAPI (blue). The left panel shows a confocal vertical section. Right panels are different planes on its *z* axis, as indicated by the vertical bars. The right panel shows maximum intensity projection images of3 to 5 z-stacks at the apical, circum-apical, and basolateral regions. Scale bars, 20 μm. Images are representative from N = 6 (2 technical replicates from 3 human donors [biological replicates]). (B) Quantification of colocalization between RNP^tracker^ and P-protein within infectious centers. Colocalization was quantified by using Mander’s colocalization coefficient. Download FIG S3, PDF file, 1.2 MB. Copyright © 2019 Singh et al. 2019 Singh et al. This content is distributed under the terms of the Creative Commons Attribution 4.0 International license.

10.1128/mBio.02434-19.8 VIDEO S2 Localization of RNP^tracker^ and N-protein in an infectious center. All confocal z-stacks of the infectious center (Fig. 5) are shown from the apical to the basolateral surface. Z-stacks of 1 μm were acquired on a Leica SPE confocal microscope. HAE cells were infected with RNP^tracker^. At 72 hpi, the cells were fixed, permeabilized, and immunostained for N protein (red). The nuclei were visualized with DAPI (blue). Download Movie S2, AVI file, 1.6 MB. Copyright © 2019 Singh et al. 2019 Singh et al. This content is distributed under the terms of the Creative Commons Attribution 4.0 International license.

10.1128/mBio.02434-19.9 VIDEO S3 Localization of RNP^tracker^ and P protein in an infectious center. All confocal z-stacks of the infectious center (Fig. S4) are shown from the apical to the basolateral surface. Z-stacks of 1 μm were acquired on a Leica SPE confocal microscope. HAE cells were infected with RNP^tracker^. At 72 hpi, the cells were fixed, permeabilized, and immunostained for P protein (red). The nuclei were visualized with DAPI (blue). Download Movie S3, AVI file, 2.2 MB. Copyright © 2019 Singh et al. 2019 Singh et al. This content is distributed under the terms of the Creative Commons Attribution 4.0 International license.

10.1128/mBio.02434-19.4 FIG S4 Localization of RNP in infectious centers. Cells were infected with MeV-RNP^tracker^ (green). At 72 hours post infection, cells were fixed and counterstained for F-actin with phalloidin (red), and nuclei visualized with DAPI (blue). The left panel shows a vertical section. Right panels are different planes on its *z* axis, as indicated by the vertical bars. The right panel shows maximum intensity projection images of three to five z-stacks at the apical, circumapical, and basolateral regions. White arrows indicate MeV RNPs along the circumapical region of the F-actin network in newly infected cells. Images are representative from *n* = 6 samples (two technical replicates from three human donors [biological replicates]). Scale bars, 10 μm. Download FIG S4, PDF file, 1.3 MB. Copyright © 2019 Singh et al. 2019 Singh et al. This content is distributed under the terms of the Creative Commons Attribution 4.0 International license.

### MeV RNPs spread to adjoining cells along the circumapical actin network.

**FIG 6:**
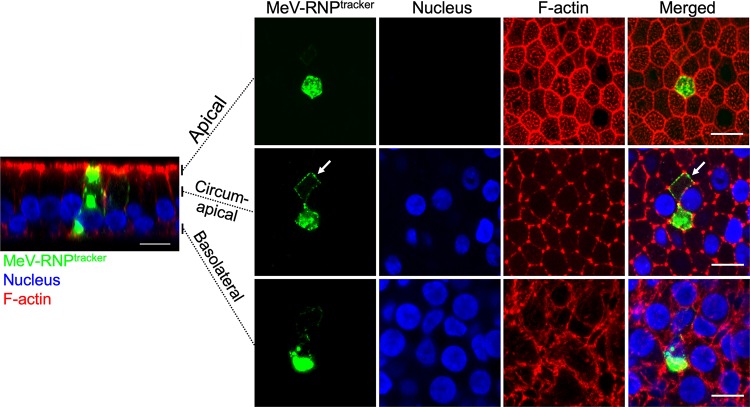
In newly infected cells, MeV RNPs localize to the circum-apical network. Cells were infected with MeV-RNP^tracker^, fixed, and imaged at 36 hpi by confocal microscopy. Cells were counterstained for F-actin with phalloidin (red), and nuclei were visualized with DAPI (blue). The left panel is a vertical section view, and the vertical bars indicate the plane of view for the series of *en face* images on the right. The right panels show maximum intensity projection images of three to five z-stacks at the indicated apical, circumapical, and basolateral regions. White arrows indicate MeV RNPs along the circumapical region of the F-actin network in newly infected cells. Images are representative from *n* = 6 samples (two technical replicates from three human donors [biological replicates]). Scale bars, 10 μm.

We previously suggested that MeV RNPs take advantage of intercellular pores and of the circumapical actin rings to rapidly spread across the human airway epithelium, a hypothesis that we can now test by MeV-RNP^tracker^-based microscopy. After infecting HAE cells with this virus, we focused on individual GFP-expressing cells at an early time point. These cells begin to appear ca. 30 to 36 h after inoculation. Remarkably, the MeV RNPs (green) encircle newly infected cells (Fig. 6, white arrow) and colocalize with F-actin (red) in the circumapical network. This colocalization of MeV RNPs and F-actin at apical and circumapical networks was also observed at later infection time points (72 hpi) at the periphery of infectious centers (Fig. S4, white arrows indicate MeV RNPs along the circumapical F-actin network in newly infected cells).

We then sought, using immunohistochemistry, to assess whether we can detect M protein in newly MeV-RNP^tracker^-infected cells. Figure S5 shows an infected cell next to a newly infected cell on its left. The vertical section of the left panel confirms that, like the P protein of MeV-nCFP (Fig. 3), the GFP-P protein of MeV-RNP^tracker^ localizes to perinuclear bodies, as well as proximal to the apical plasma membrane. M protein is only detected at the plasma membrane. Within the apical region, M and P localization only partially overlaps, with the bulk of M-protein closer to the plasma membrane than the P protein. The horizontal sections on the right indicate that both the GFP-P and the M proteins of MeV-RNP^tracker^ transfer to the newly infected cell.

10.1128/mBio.02434-19.5 FIG S5 Localization of RNP^tracker^ and M protein in infectious centers. Cells were infected with MeV-RNP^tracker^ (green). At 36 hpi, the cells were fixed and colabeled using anti-matrix antibodies (red), and nuclei were visualized with DAPI (blue). The left panel shows a confocal vertical section. Right panels are different planes on its *z* axis, as indicated by the vertical bars. The right panel shows maximum intensity projection images of three to five z-stacks at the apical, circumapical, and basolateral regions. Green arrows indicate MeV RNPs along the apical, circumapical, and perinuclear regions. The red arrows indicate M-protein. Images are representative from *n* = 6 samples (two technical replicates from three human donors [biological replicates]). Scale bars, 10 μm. Download FIG S5, PDF file, 0.8 MB. Copyright © 2019 Singh et al. 2019 Singh et al. This content is distributed under the terms of the Creative Commons Attribution 4.0 International license.

**FIG 7:**
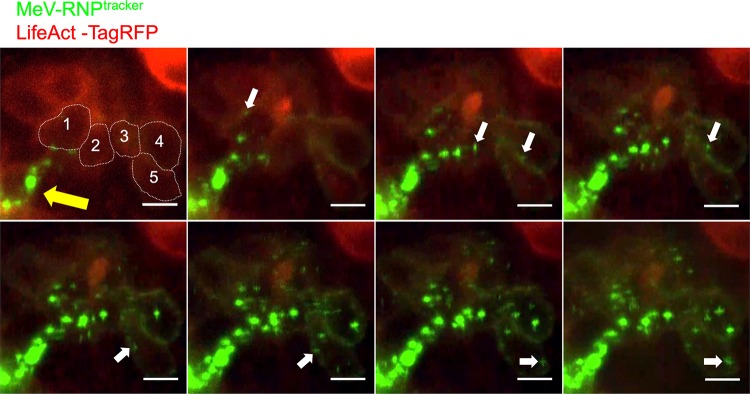
Time-lapse microscopy analysis of MeV RNPs transport along the F-actin network. HAE cells were transduced with an adenoviral vector expressing LifeAct-TagRFP (red). After 24 h, HAE cells were infected with MeV-RNP^tracker^ (green). Fluorescence was monitored by confocal time-lapse microscopy beginning at 48 h after MeV infection; the spread of MeV-RNPs is shown at approximately 2-h intervals. The yellow arrow indicates the initial infected cell. Dotted lines in the first panel indicate individual columnar epithelial cells, as well as the circumapical F-actin networks. The cells are numbered 1 to 5 from nearest to furthest from the initial infected cell. White arrows indicate the unidirectional flow of MeV RNPs. Images correspond to Video S4. Scale bars, 10 μm.

To further investigate the cell-to-cell spread of MeV RNPs, we monitored MeV-RNP^tracker^ spread in HAE cells by live-cell microscopy. For visualization of F-actin in live cells, HAE cells were transduced with an adenoviral vector expressing TagRFP-tagged LifeAct, a 17-amino-acid peptide that binds to F-actin in live cells without disruption of cellular processes (45). After 24 h, the cells were infected with MeV-RNP^tracker^. Cells coexpressing TagRFP and eGFP were selected for live cell microscopic imaging over a 16-h time period (48 to 64 h after MeV infection) (Fig. 7 and Video S4). Over the 16-h time course, numerous individual eGFP puncta appeared at the plasma membrane and gradually increased in fluorescence intensity, likely from the recruitment of additional RNP complexes. White arrows show the location of eGFP puncta as it moves unidirectionally along the circumapical F-actin (Fig. 7 and Video S4). This observation was reproducible in multiple infectious centers. Our conclusions concerning the cell-to-cell movement of MeV-RNP^tracker^ are based on three movies from HAE derived from two donors. Of note, the timing and the rate of spread in this experiment are consistent with our previous observations of the direct cell-to-cell spread of MeV-expressed cytosolic GFP (24).

10.1128/mBio.02434-19.10 VIDEO S4 MeV RNPs spread from cell-to-cell along the F-actin network. HAE were transduced with an adenoviral vector expressing LifeAct-TagRFP (red) and, 24 h later, with MeV-RNP^tracker^ (green). Time-lapse confocal microscopy began 48 h after MeV infection. A total of 50 confocal z-stacks at 1-μm intervals were acquired every 10 min over a period of 16 h. The movie is a maximum intensity projection of 10 z-stacks at circumapical F-actin network region and played at 10 frames per second. The cells are numbered 1 to 5 from nearest to farthest from the initial infected cell. A unidirectional flow of MeV RNPs from the nearest to furthest cells can be seen along the F-actin network. The movie corresponds to the series of images in Fig. 7. Scale bar, 10 μm. Download Movie S4, MOV file, 3.7 MB. Copyright © 2019 Singh et al. 2019 Singh et al. This content is distributed under the terms of the Creative Commons Attribution 4.0 International license.

## DISCUSSION

We characterized MeV spread in well-differentiated, primary cultures of human airway epithelial cells. Based on a newly generated RNP “tracker” virus, we documented by video microscopy that RNPs move along the circumapical F-actin ring and enter a new cell. Thus, rather than diffusing through the cytoplasm of a newly infected columnar cell, RNPs take advantage of the cytoskeletal infrastructure to spread laterally across HAE. This results in rapid horizonal spread through the epithelium, as predicted by the intercellular-pore/fast-lane model (21, 24).

### Viral spread through intercellular pores versus cell fusion.

MeV infections have been studied mainly in monolayers of immortalized cells, where the virus spreads through cell fusion. Receptors on recipient cells trigger the viral membrane fusion apparatus on the surfaces of infected cells to form fusion pores. Rapid pore expansion results in the coalescence of plasma membranes and formation of multinucleated syncytia. However, in the airways of living hosts, syncytia are rarely detected (46–48).

Lack of syncytium formation in response to MeV infection is recapitulated in HAE cells, where large infectious centers are formed, while transepithelial resistance is maintained (23). Furthermore, plasma membranes of individual infected cells remain intact, suggesting that fusion pores do not expand (23, 24). Even in the absence of cytopathology, MeV spread in the human airway epithelium is more rapid than that of other respiratory viruses (24). Thus, while extensive cell fusion occurs in infected monolayers of transformed cells, it may be counterproductive for infections of wild-type viruses in natural hosts.

### F-actin and viral replication.

We document how RNPs traveling on circumapical F-actin rings traverse individual columnar cells and cross over to the next one. During this process, some RNPs may be diverted from the apical rings and transported to a perinuclear location, where replication centers form. These RNPs could be inactive when they reach the adherens junction of the originally infected cell and become transcriptionally active in the new host cell. Alternatively, small, transcriptionally active replication centers could be transported intercellularly. In both cases, an almost synchronous wave of RNP infiltration would follow the opening of an intercellular fusion pore. This synchronous infection with multiple viral genomes may overwhelm the antiviral defenses of naive cells.

We consistently observe that the lateral regions of the F-actin network break down within the innermost cells of infectious centers but remains intact within peripheral cells. While the mechanistic cause of apical F-actin ring breakdown remains unclear, this process may favor directional movement of MeV RNPs from infected to uninfected cells, thereby preventing superinfection. In addition, disassembly of the circumapical network may redirect traversing RNPs to perinuclear locations most favorable for their replication.

Different observations suggest multiple roles for F-actin in MeV replication. F-actin was observed in close association with MeV RNPs in infected cells (40), and it was also reported that inhibitors of actin polymerization restrict MeV replication (49, 50). Intriguingly, other studies documented large amounts of monomeric actin in MeV particles (36, 51, 52), far exceeding actin levels reported for other paramyxovirus particles (53). Although immune cells lack the same defined cytoskeletal organization of well-differentiated columnar epithelial cells, our observations may be consistent with how MeV spreads between monocytes and/or lymphatic cells. Indeed, MeV transmission from dendritic cells to lymphocytes relies on the formation of transient adhesive structures similar to virological synapses, where F-actin accumulates at the cellular junctions (54), again implicating F-actin in virus transmission. Future studies using RNP^tracker^ and live-cell imaging of MeV-infected primary monocytes may further elucidate how it spreads systemically. Extensive colocalization of MeV RNPs to the circumapical F-actin network during infectious center formation in the human airway epithelium is consistent with previous observations and provides a mechanism for rapid cell-to-cell spread. Altogether, these observations are consistent with transport of MeV components on F-actin.

Our previous study highlighted the importance of the Nectin-4/Afadin complex during MeV spread in HAE (24), but thus far it remains unclear which F-actin binding and signaling proteins facilitate the transport of RNPs along the circumapical F-actin network. Additional cellular proteins are likely involved in RNP transport from infected to uninfected cells, as well as in switches between actin filaments (55, 56).

### Mechanisms of intra- and intercellular virus spread.

Microtubules are assumed to be the major cellular transporter highways (55). However, recent studies highlighted the importance of actin-dependent transport of Rab11-containing vesicles with several different cargos toward the plasma membrane of mammalian cells (57). Indeed, microtubule dependent Rab11A-positive endosomes mediate intracellular transport of MeV RNP complexes in Vero/hSLAM cells and polarized epithelial cells (58). A combination of microtubule- and actin-dependent movement may be responsible for intra- and intercellular transport of MeV RNP complexes.

Using HAE cells, we previously characterized intercellular pores that allow the transfer of cytoplasmic contents from MeV-infected to naive neighboring columnar epithelial cells (24, 37). Here, we address the critical question of what traffics through the pores. Opening membrane pores in HAE cells likely requires viral H and F proteins, implying that these proteins travel with the RNP. The M protein may be critical for bridging RNPs with the F and H proteins. Our data suggest that M protein also rapidly reaches naive cells but does not exclusively colocalize with RNPs. As such, partially assembled MeV particles composed of different combinations of viral proteins may spread cell to cell in HAE cells.

For viruses of several different families, a high concentration of components at sites of cell-cell contact may account for cell-to-cell transmission being more efficient than particle-dependent transmission (18–20). In polarized cells of the respiratory epithelium, MeV components are concentrated mainly to the narrow cytoskeletal ring connected to the adhesive and tight junctions and located near the apical surface. This concentration mechanism permits spreading at much reduced viral and cellular costs, allowing lower viral gene expression and extending cell viability. Other viruses use variations of this strategy. For example, in the case of HIV, particles on the cell surface are likely drawn to the virological synapse through cytoskeletal interactions (59, 60). In the case of poxviruses, cytoskeletal structures forming below the cell surface push particles to “surf” toward noninfected cells (61). Herpes viruses spread in polarized neurons by a virus-directed mechanism that operates by coordinating opposing microtubule motors to favor sustained retrograde delivery of the virus to the peripheral ganglia (62). Thus, viruses can utilize existing sites of cell-cell contact, such as adhesive and tight junctions or neurological or immunological synapses, to most efficiently spread in the host.

In conclusion, our studies further develop a paradigm for the rapid intrahost spread of viruses that relies on cell-cell adhesion and on cytoskeletal driving forces and is particle independent. Such cell-associated mechanisms often result in the cotransmission of multiple viral genomes, further enhancing the efficacy of virus dissemination within hosts (63).

## MATERIALS AND METHODS

### Ethical statement.

Primary cultures of human airway epithelial (HAE) cells were prepared from discarded tissue, autopsy, or surgical specimens. All specimens used in this study are obtained from the University of Iowa In Vitro Models and Cell Culture Core Repository. We were not provided with any information that could be used to identify a subject. All studies involving human subjects received University of Iowa Institutional Review Board approval.

### Recombinant MeV used and generation of MeV-RNP^tracker^.

All four recombinant MeV used in this study are derivatives of wild-type strain IC-323 (64, 65). The generation of MeV-GFP (65) and MeV-nCFP (42) were previously published. MeV-RNP^tracker^ and its control virus MeV(GFP)H were generated for this study. MeV production was conducted as previously described (65, 66). The design of MeV-RNP^tracker^ is based on a previous study showing that a vaccine-lineage MeV expressing a GFP-P chimeric protein can replicate to high titers, provided that the original copy of the P gene is maintained (67). To generate a eGFP-P fusion construct, the P open reading frame was amplified from the p(+)MeV323 plasmid (64) using the primers P_323_-fwd (GTA CCG GTG GAG CAG AAG AGC AGG CAC GCC ATG) and P_323_-rev (TTG CAT GCG CTA CTT CAT TAT TAT CTT CAT CAG CAT CTG GTG G) and cloned into pCG-eGFP-P_NSe_ (67) using AgeI and SphI restriction sites, replacing the P protein of the vaccine-lineage strain (P_NSe_). To express only GFP-P, without expressing GFP-V, the polypurine tract required for G-nucleotide insertion was mutagenized (68) using the primers P_323_-V^KO^-fwd (GAC ACC CAT TAA AGA GGG CAC TGA CGC GAG ATT GGC CTC ATT TG) and P_323_-V^KO^ (AAT GGG TGT CTC GGA AGT GCT GG) to generate pCG-eGFP-P_323_[V^KO^]. The modified open reading frame was then PCR amplified using the primers eGFP-P_323_-fwd (ATA TAT GAA TTC ACG CGT ACG ATG GTG AGC AAG GGC GAG) and eGFP-P_323_-rev (TAT ATA CTC GAG GAC GTC CTA CTT CAT TAT TAT CTT CAT CAG CAT CTG G) and cloned into p(+)MeV323(GFP)H (C. S. A. Ferreira, unpublished data) using the restriction sites MluI and AatII to generate p(+)MeV323(eGFP-P_323_[V^KO^])_H,_ replacing the eGFP open reading frame with the eGFP-P_323_[V^KO^] open reading frame. MeV-RNP^tracker^ virus was rescued from p(+)MeV323(eGFP-P_323_[V^KO^])_H_, and viral stocks were generated and titrated as previously described (66).

### MeV production and titering.

MeV production was conducted as previously described (65, 66). Briefly, Vero cells stably expressing the MeV receptor SLAMF1 (Vero-hSLAM cells, kindly provided by Y. Yanagi) (69) were cultured in Dulbecco modified Eagle medium (DMEM; Thermo Fisher Scientific) containing 8% newborn calf serum (NCS; Thermo Fisher Scientific) and penicillin-streptomycin (100 mg/ml; Thermo Fisher Scientific) were used to produce MeV. Recombinant MeV titers of approximately 10^7^ 50% tissue culture infective doses (TCID_50_)/ml were obtained.

### Western blot analysis.

For Western blot analysis, 10^6^ Vero-hSLAM cells (8) were either infected with MeV-eGFP or MeV-RNP^tracker^ at a multiplicity of infection (MOI) of 0.1 or left uninfected and then harvested at 24 hpi and lysed in 300 μl of denaturing protein lysis buffer (62.5 mM Tris [pH 6.8], 2% [wt/vol] sodium dodecyl sulfate, 10% [vol/vol] glycerol, 6 M urea, 5% [vol/vol] β-mercaptoethanol, 0.01% [wt/vol] bromphenol blue). Samples were heated at 95°C for 10 min and 10 μl/lane were loaded on 10% SDS-PAGE gels. Western blots were performed as previously described (44). The antibodies used for detection were polyclonal rabbit anti-N_505_ (1:5,000 [70]), polyclonal rabbit anti-P_254_ (1:5,000 [70]), monoclonal mouse anti-GFP (clone GF28R, 1:1,000, catalog no. 14-6674-82 [Thermo Fisher Scientific]), horseradish peroxidase (HRP)-conjugated mouse monoclonal anti-β-actin (1:25,000, catalog no. A3854-200UL [Sigma-Aldrich]), HRP-conjugated goat anti-mouse IgG(H+L) (1:10,000, catalog no. 401215 [Millipore Sigma]), and HRP-conjugated goat anti-rabbit IgG(H+L) (1:25,000, catalog no. 111-035-144 [Jackson ImmunoResearch]).

### Primary HAE cells.

HAE cells were collected from tracheas and bronchi by enzymatic dispersion using established methods (22). Briefly, epithelial cells were dissociated and seeded onto collagen-coated, semipermeable membranes (0.4-μm pore size; surface area, 0.33 cm^2^; Corning, Corning, NY). HAE cultures were maintained in Ultraser G (USG) media at 37°C and 5% CO_2_. Polyester transwell inserts were placed into 24-well plastic cell culture plates (Costar, Cambridge, MA). At 24 h after seeding, the mucosal medium was removed, and the cells were allowed to grow at the air-liquid interface as reported previously (22). Only well-differentiated cultures (>3 weeks old) were used in these studies. The presence of tight junctions was confirmed by measuring the transepithelial resistance using a volt-ohm meter (World Precision Instruments, Sarasota, FL; resistance, >500 Ω⋅cm^2^).

### Infection of primary airway epithelial cells.

Infection of HAE cells was performed as previously described (23, 24). Briefly, to infect airway epithelia with MeV from the basolateral side, the cultures were inverted, and the virus was applied to the basolateral surface for 4 h in 80 μl of serum-free medium. After the infection, the virus was removed, and the transwell insert was turned upright and incubated at 37°C under 5% CO_2_ for the indicated times. For actin visualization in time-lapse microscopy, HAE cells were transduced with adenovirus-containing LifeAct-TagRFP (Ibidi, Munich, Germany) at a preoptimized MOI according to the manufacturer’s protocol.

### Inhibitor treatment.

Inhibitor stocks were prepared in DMSO at concentrations of 4 mM for CytoD (Sigma-Aldrich), 10 mM for CK-666 (Sigma-Aldrich), and 3 mM for SMIFH2 (Sigma-Aldrich). Different dilutions of the inhibitors were assessed (1 to 4 μM for CytoD, 100 to 500 μM for CK-666, and 100 to 500 μM for SMIFH2) to determine the appropriate working concentrations that have maximal effects on the actin cytoskeleton (see Fig. S6 in the supplemental material) without causing a loss of transepithelial resistance below 500 Ω·cm^2^. At 36 h after MeV-GFP infection, HAE cells were treated apically and basolaterally with final concentrations of 4, 500, and 100 μM for CytoD, CK-666, and SMIFH2, respectively, for 24 h. After treatment, the HAE cells were imaged using an inverted UV fluorescence microscope. The data are presented as means ± the standard deviations of the log-transformed value of individual data points. Statistical significance as adjusted *P* values between groups was determined by using one-way analysis of variance (ANOVA) with corrections for multiple comparisons.

10.1128/mBio.02434-19.6 FIG S6 Effect of cytoskeletal disrupting drugs on F-actin network. Cytochalasin D (CytoD), CK-666, SMIFH2, or control (DMSO) was applied to the apical and basolateral surfaces for 24 h. Cells were fixed, permeabilized, and counterstained for F-actin using phalloidin (green). Nuclei were visualized with DAPI (blue). Images are representative from *n* = 6 samples (two technical replicates from three human donors [biological replicates]). Scale, 20 μm. Download FIG S6, PDF file, 1.8 MB. Copyright © 2019 Singh et al. 2019 Singh et al. This content is distributed under the terms of the Creative Commons Attribution 4.0 International license.

### Immunostaining and microscopy.

At the indicated times postinfection, HAE cultures were processed for confocal microscopy. For processing, the cells were fixed in 2% paraformaldehyde, permeabilized in 0.2% Triton X-100, and blocked in 1% Superblock (Thermo Fisher Scientific) for 1 h. For immunostaining, HAE cells were incubated with the primary antibodies anti-N_505_ (1:200 [24]), polyclonal rabbit anti-P_254_ (1:200 [44]), and anti-M (1:200, catalog no. MAB8910 [Millipore-Sigma]) overnight at 4°C. Secondary antibodies were either Alexa 568-labeled goat anti-mouse and Alexa 568-labeled goat anti-rabbit secondary antibody (Life Technologies) for confocal imaging or Alexa 532-labeled goat anti-mouse and Alexa 532-labeled goat anti-rabbit secondary antibody (Life Technologies) for STED imaging. Actin was stained with either rhodamine-phalloidin (1:100, catalog no. R415; Thermo Fisher Scientific) or phalloidin-Alexa 647 (1:40, catalog no. A22287; Thermo Fisher Scientific) for 30 min. The transwell inserts containing the cells were separated from the plastic cylinder by cutting the edges with a razor blade. The cells were mounted on a slide with either Vectashield with DAPI (4′,6′-diamidino-2-phenylindole; Vector Laboratories, Inc., Burlingame, CA) for confocal imaging or with ProLong Diamond with DAPI antifade reagent (catalog no. P36971; Thermo Fisher Scientific) for STED imaging. Unless otherwise noted, immunostaining was performed on at least two cultures (technical replicates) from three human donors (biological replicates) for all microscopy figures. Images were captured from each donor, and representative images are shown. We note that the sizes and numbers of infectious centers varied from donor to donor; however, the expression pattern of viral proteins and F-actin remained remarkably consistent.

Confocal images were acquired on a Leica TCS SP3 confocal microscope (Leica Microsystems, Inc., Buffalo Grove, IL) using a 40× or 63× oil immersion objective. STED images were acquired on a Leica SP8 STED 3× (Leica Microsystems, Inc.) platform equipped with lasers for the depletion of fluorophores emitting in the orange (660 nm; Laser Quantum) and red/far red (775 nm; OneFive, GmbH) using a 100× NA:1.4 oil immersion lens objective. Confocal and STED images were obtained at 1,024 × 1,024 or at 2,048 × 2,048 pixels, respectively. A pair of depletion wavelength lasers (nm = 660/592) or a single wavelength laser (nm = 660) was applied for multicolor STED imaging. To optimize resolution without bleaching, the STED lasers were applied at the lowest power that can provide sufficient improvement in resolution compared to confocal imaging. STED images were deconvolved with Huygens essential software (Scientific Volume Imaging) using the CMLE algorithm. We analyzed 50 to 60 optical z-stacks (step size, 1 μm) using ImageJ 1.47v software.

To determine the colocalization of red and green signals, the images were analyzed as z-stacks using channel 1 for green and channel 2 for red. The region of interest was analyzed using the Coloc2 plugin with the Costes method for automatically estimating threshold values for identifying background levels (71, 72).
