# Supplementary figures and images for: Measles Virus Ribonucleoprotein Complexes Rapidly Spread across Well-Differentiated Primary Human Airway Epithelial Cells along F-Actin Rings
Source: mBio. 2019 Nov 26;10(6):e02434-19. doi: 10.1128/mBio.02434-19 (PMC6879720; doi:10.1128/mBio.02434-19)

Figure S1

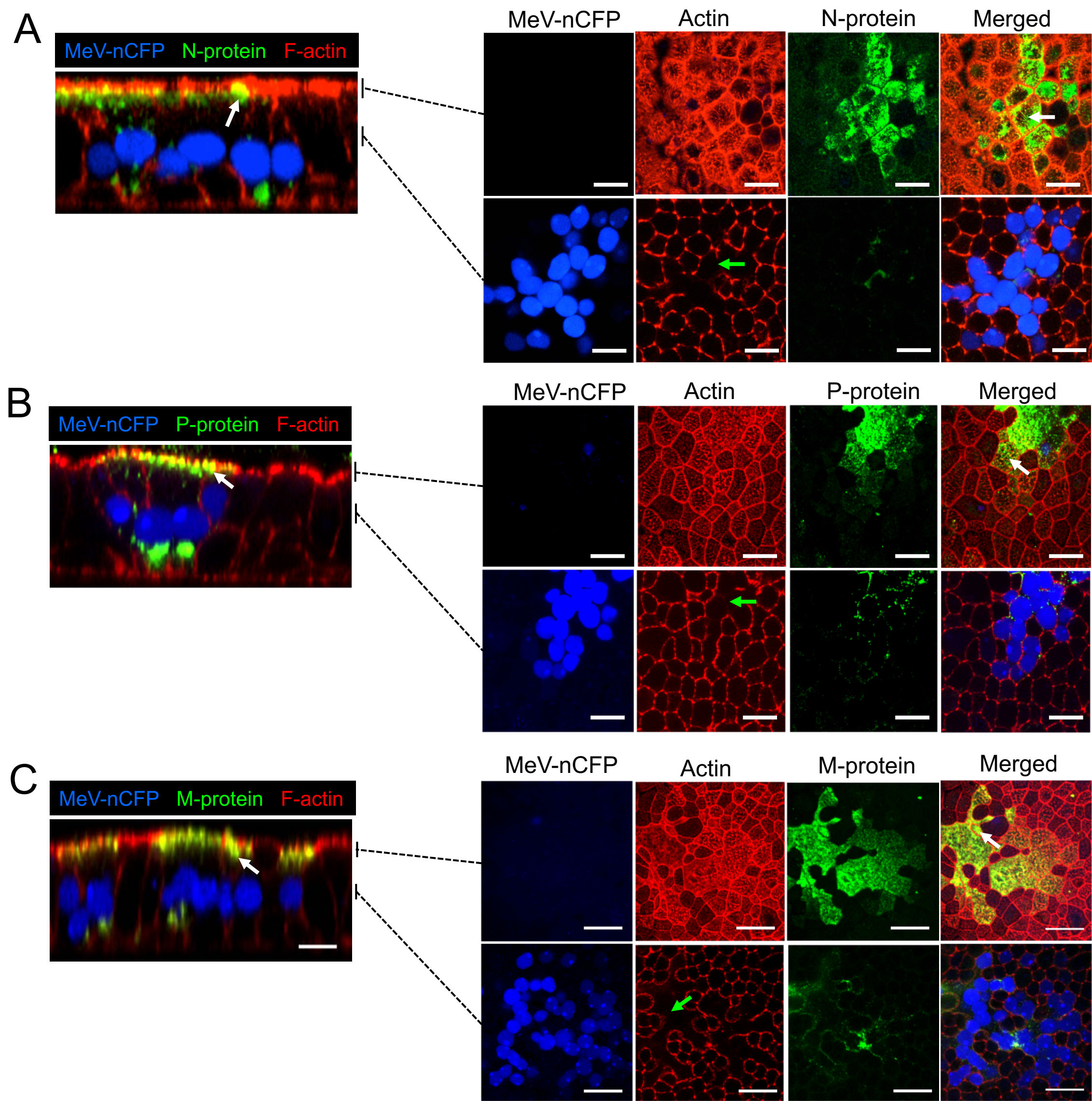

Supplement: FIG S1 [file mBio.02434-19-sf001.pdf]

Figure S2

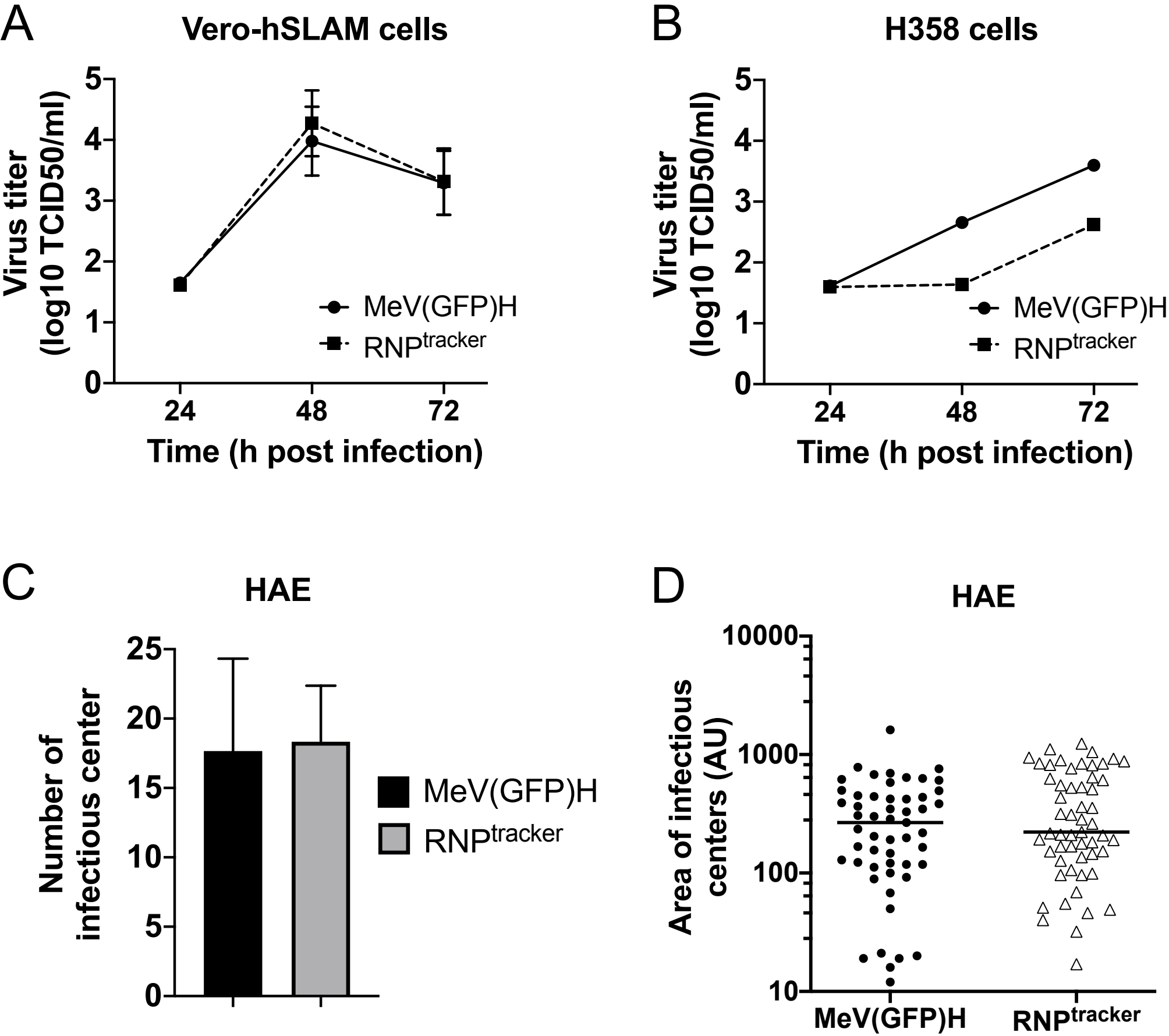

Supplement: FIG S2 [file mBio.02434-19-sf002.pdf]

Figure S3

A

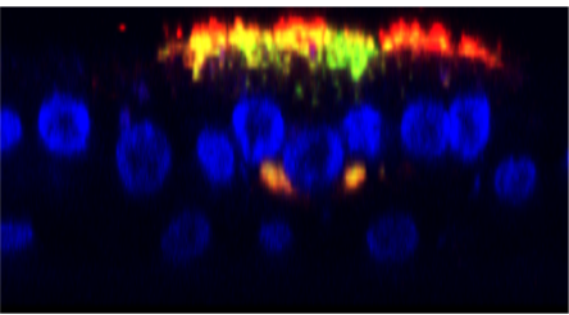

B

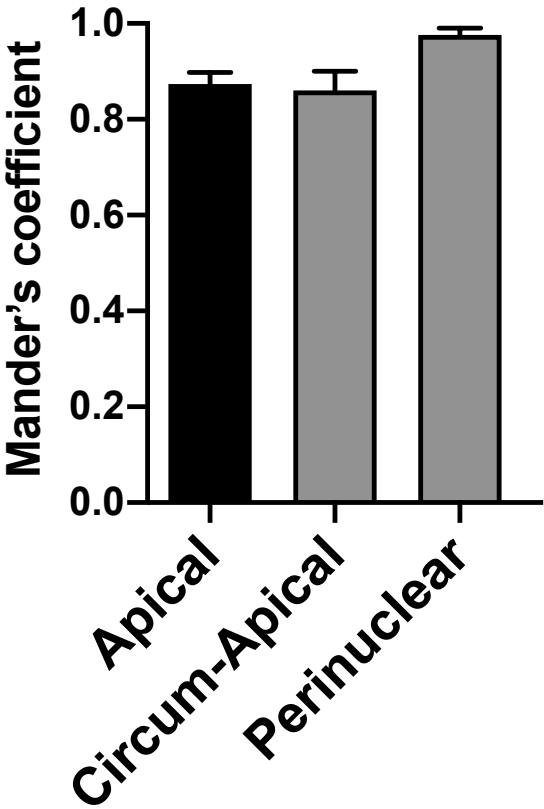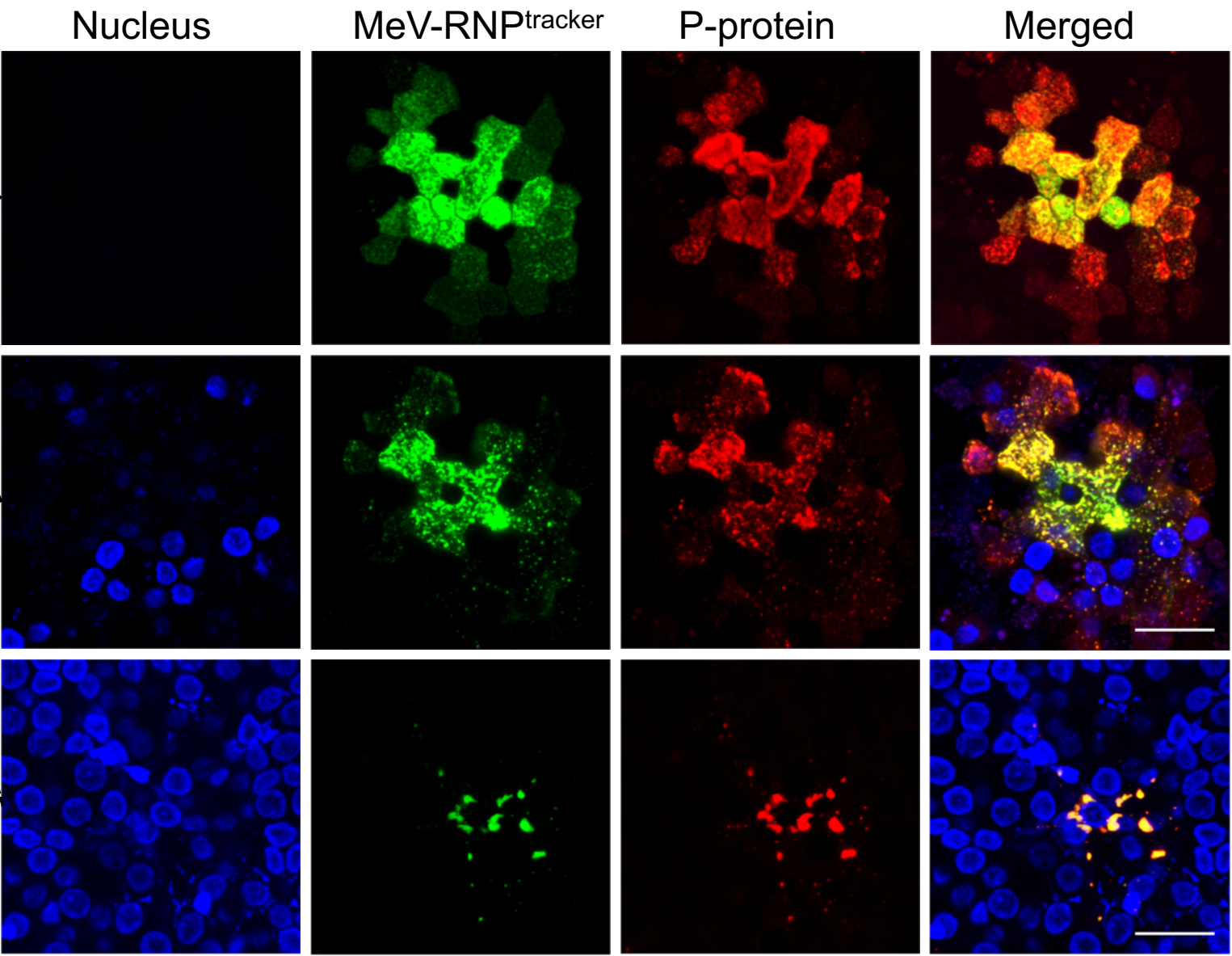

Supplement: FIG S3 [file mBio.02434-19-sf003.pdf]

Figure S4

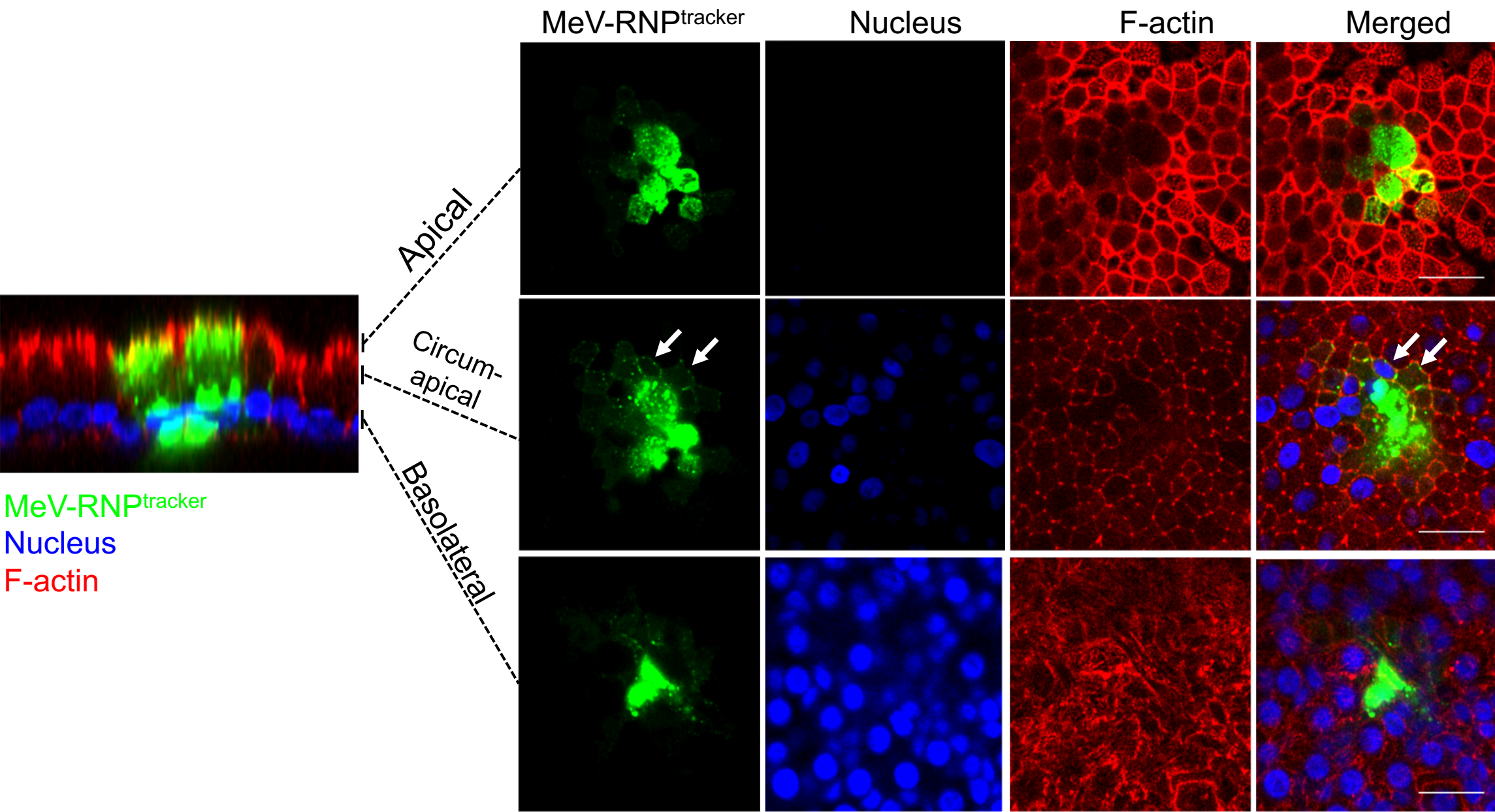

Supplement: FIG S4 [file mBio.02434-19-sf004.pdf]

Figure S5

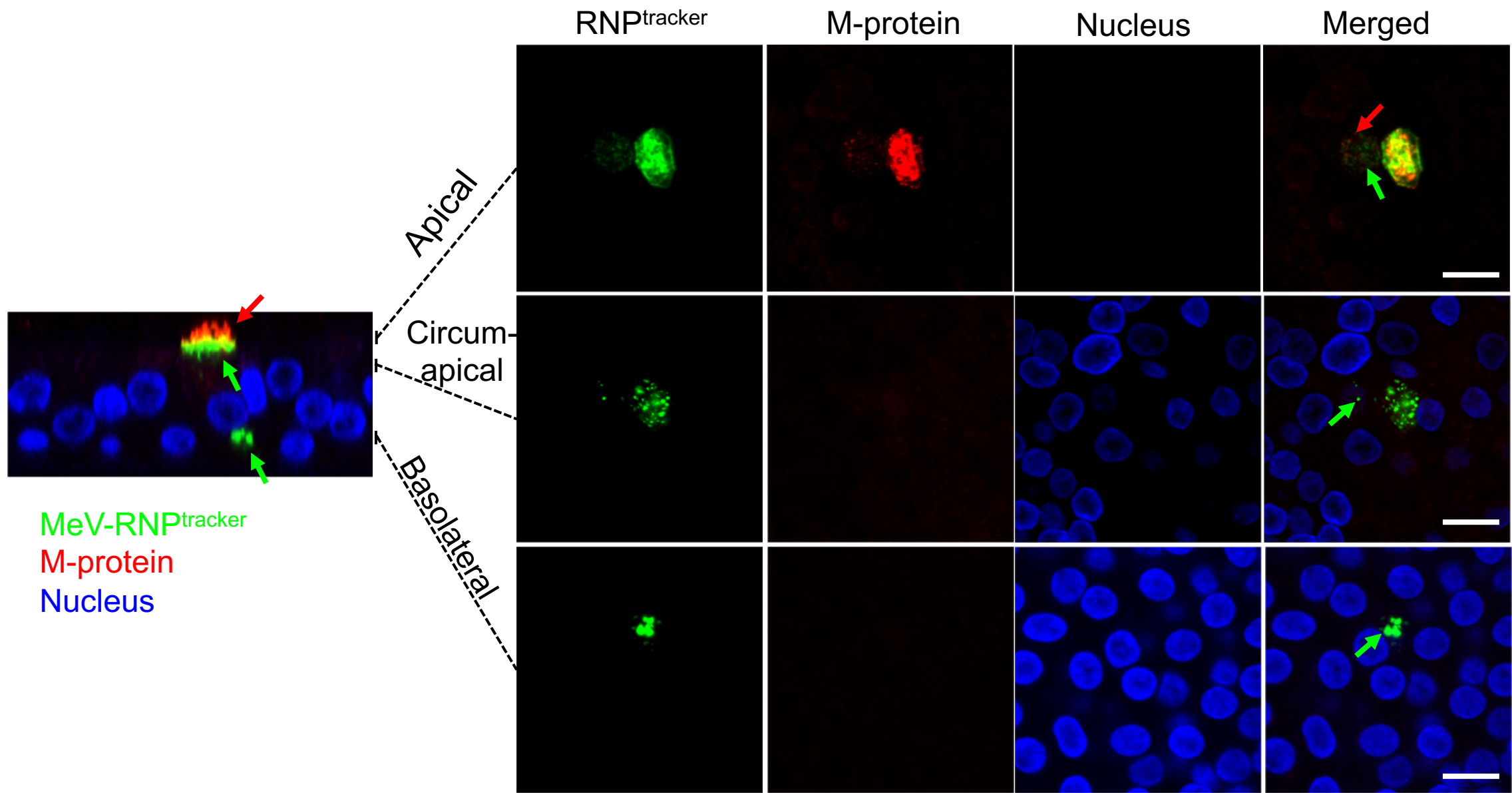

Supplement: FIG S5 [file mBio.02434-19-sf005.pdf]

Figure S6

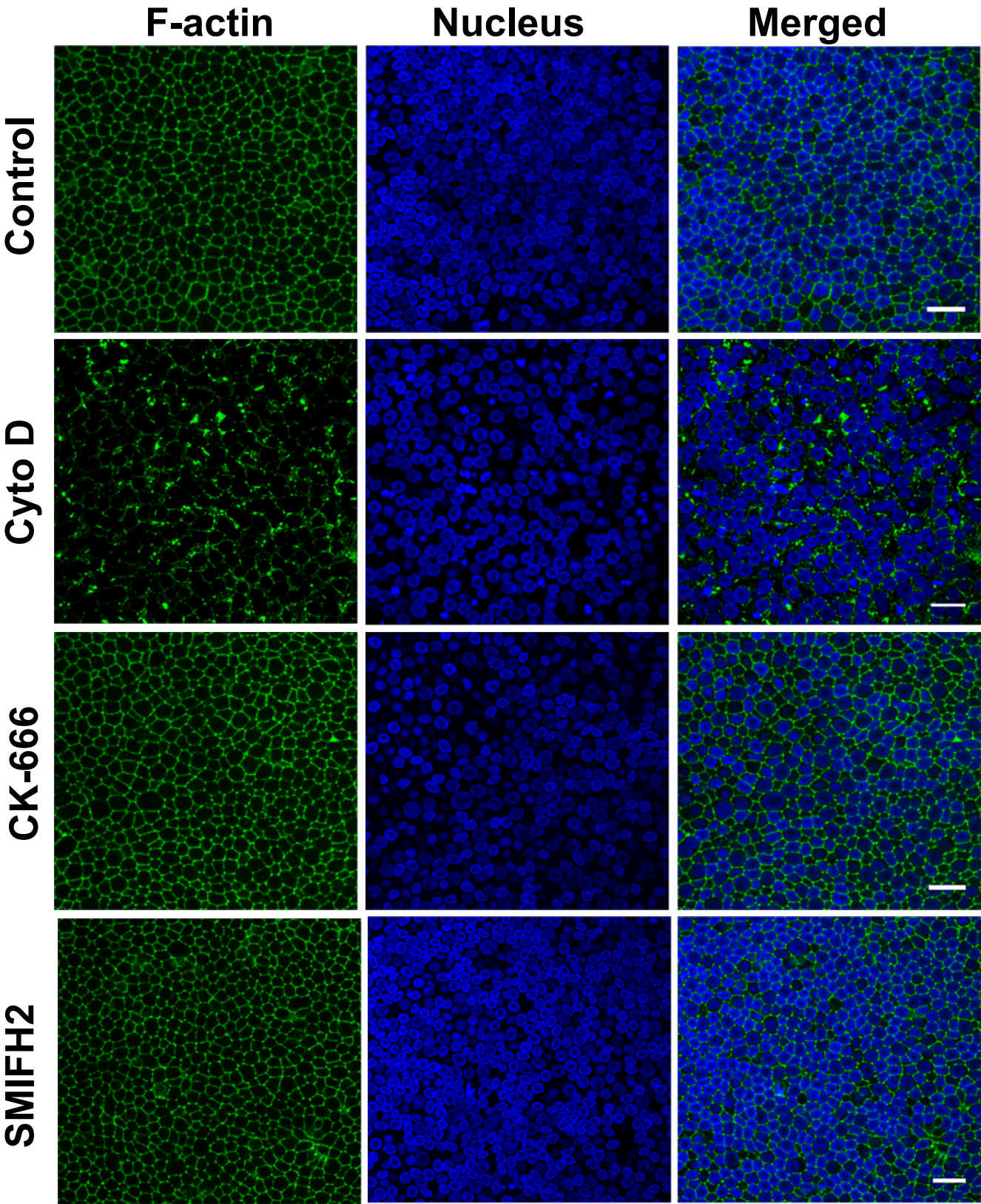

Supplement: FIG S6 [file mBio.02434-19-sf006.pdf]
